# Regeneration and Reuse of Immunoaffinity Column for Highly Efficient Clean-Up and Economic Detection of Ochratoxin A in Malt and Ginger

**DOI:** 10.3390/toxins10110462

**Published:** 2018-11-08

**Authors:** Xi Liu, Xiaofei Liu, Pinxuan Huang, Fang Wei, Guangyao Ying, Jinghua Lu, Lidong Zhou, Weijun Kong

**Affiliations:** 1 Pharmacy College, Jinzhou Medical University, Jinzhou 121001, China; liuxi119@sohu.com (X.L.); xuanpinh@126.com (P.H.); fangwei161@sina.cn (F.W.); guangyaoy88@163.com (G.Y.); 2 Institute of Medicinal Plant Development, Chinese Academy of Medical Sciences & Peking Union Medical College, Beijing 100193, China; feifeill@163.com

**Keywords:** ochratoxin A, immunoaffinity column, regeneration and reuse, malt and ginger, HPLC-FLD

## Abstract

Immunoaffinity columns (IACs) are most popularly used for mycotoxin clean-up in complex matrices prior to chromatographic analysis. But, their high cost has limited their wide application and the regeneration of IACs for multiple instances of reuse is important. This study aimed to investigate the feasibility of regeneration and reuse of IACs for purification of ochratoxin A (OTA) in spiked raw malt and dried ginger samples followed by high performance liquid chromatography-fluorescence detection. After each use, the IACs were filled with phosphate buffer saline (PBS) as the preservation solution and stored at 8 °C overnight for regeneration and reuse until the recovery rate was <70%. The results showed that matrix type, preparation procedure, and pH value of sample extraction exhibited major effects on the reuse of IACs for OTA clean-up. While, after modifying the sample preparation procedure using water as the diluent and the solution at a pH of 7 to 8, the IACs could be used eight and three times for the spiked raw malt and dried ginger samples with OTA after regeneration. Regarding the traditional procedure recommended in Chinese Pharmacopoeia (2015 edition), the IACs could be used for three and two times for the spiked raw malt and dried ginger samples with OTA, respectively. Therefore, the corresponding experimental cost could be reduced to one-eighth and one-third of the original cost. This is the first study on the regeneration and reuse of IACs for OTA clean-up in complex Chinese herbal medicines, providing a green and economical tool for a large number of samples analysis with low cost.

## 1. Introduction

Chinese herbal medicines (CHMs), cereals, and subsidiary agricultural products are susceptible to mycotoxins under the high temperature and humidity conditions during their growth, harvesting, processing, and storage processes [1,2,3]. As the toxic secondary metabolites produced by toxigenic fungi, mycotoxins exhibit serious toxicity to human and animals. Among them, ochratoxin A (OTA), mainly produced by certain Asperillus and Penicillium strains, is one of the most problematic mycotoxins [4]. Due to its serious nephrotoxicity, carcinogenicity, teratogenicity, mutagenicity, and immunosuppressive effects [5], OTA has been classified as possibly carcinogenic to humans (group 2B) by the International Agency for Research on Cancer (IARC) [6]. Owing to reproductive and developmental toxicity to the humans and other organisms, the Joint FAO/WHO Expert Committee on Food Additives (JECFA) has established the provisional tolerable weekly intake level of OTA at 100 ng/kg body weight [7].

In recent years, OTA has been found in a large number of CHMs, including malt and ginger [8,9,10,11] that have been widely used as valuable medicinal and edible CHMs or foods, in the world with various pharmacologic actions and nutritional functions. The reported incidence of OTA in malt is up to 12.5%, which will affect the quality and safety, as well as lower the medical and edible values of this CHM/food. More importantly, OTA could be detected not only in the peel, but also in the innermost layer of ginger, although no significant mildew was observed on the surface by naked eye. This is an extremely serious fact, posing a potential threat to the consumers of ginger and related products. Taking the high incidence and toxicity into consideration, WHO recommends that the highest permitted residue level of OTA in cereals is 5.0 μg/kg. The European Union restricted that the maximum amount of OTA in infant foods for medical purposes is 0.5 μg/kg [12]. In 2003, China also stipulated that the OTA limit for cereals and beans should not exceed 5.0 μg/kg [13], while, no limits are listed for OTA in CHMs from then on.

A large number of analytical methods, such as chemical techniques [14], chromatographic [15], electrochemical [16], and immunoassay methods [17] have been developed to detect OTA in foods, feeds, and CHMs. They all have their own advantages and disadvantages. Considering the complex compositions and possible interferences of CHM samples, immunoaffinity column (IAC) clean-up, to remove matrix interferences for achieving effective purification and enrichment of OTA, is the preferred technique prior to chromatographic analysis. By coupling a certain amount of monoclonal antibody of a given mycotoxin on a suitable support, the target mycotoxin in the extract will be bound by its specific antibodies on the column when the sample solutions flow through the column. After the removal of water-soluble impurities remaining on the column with water or buffer solution, the target mycotoxin is eluted from the IAC with an organic solvent such as pure methanol or acetonitrile for the following detection. As a universal and valid cleanup tool for trace analytes such as mycotoxins, IACs exhibit the advantages of high sensitivity, selectivity, and recovery, being user-friendly and solvent-saving because of the specificity of antibodies [18]. However, different matrix solutions and the addition of organic solvents will denature or devitalize the antibodies, leading to difficulty in the reuse of IACs. If the contents of target mycotoxins in the sample extract exceed the column binding capacity, the excess toxin will not be effectively bound or captured, resulting in unreliable results. In addition, IACs are known to be more expensive than other cleanup devices or cartridges for sample purification. For multiple targets the price will be even higher, which is a huge obstacle for general laboratories and limits their real application.

Bearing in mind the current situations, in recent years, studies on the regeneration of IACs have been posed. Boudra et al. [19] reported that the regenerated IACs for OTA in milk could be reused more than three times without loss binding properties. Rhemrev et al. [20] developed a new type of reusable immunoaffinity cassette that could be used for 15 times for fully-automated aflatoxin analysis with satisfactory recovery rates. Iha et al. [21] worked on the regeneration of IACs for aflatoxins that could be utilized up to nine times in peanut confection. Obviously, these reported studies are mainly focused on food matrices and CHMs were not involved.

Taking the high application frequency and cost of IACs as well as the high incidence of OTA in CHMs into account, it is of great importance to develop a protocol for IAC regeneration in order to minimize the expenses and extend the IAC-based analytical method. Therefore, this study aimed to evaluate the performance of the multitime-regenerated IACs for high performance liquid chromatography-fluorescence detection (HPLC-FLD) of OTA in the spiked raw malt and dried ginger samples. The development of a reproducible IAC will be beneficial to save cost for OTA detection of a range of CHMs, and to prevent harm to the consumer caused by consumption of high levels of OTA-contaminated CHMs. To the best of our knowledge, this is the first study on the regeneration and reuse of IACs for highly efficient clean-up and economic detection of OTA in CHMs.

## 2. Results

### 2.1. HPLC-FLD Method Validation

For accurate quantitation of OTA in the spiked raw malt and dried ginger samples, the HPLC-FLD method was firstly validated regarding selectivity, linearity, sensitivity, limit of detection (LOD), limit of quantification (LOQ), precision, and accuracy.

The selectivity of the method was evaluated after using the above-mentioned sample preparation and IAC cleanup procedure. Under the optimized chromatographic conditions, no interference peaks were observed at the retention time (4.942 min) of OTA, demonstrating good selectivity of the established HPLC-FLD method.

In regard to the linearity of the developed method, a series concentration of OTA working solutions were prepared by diluting the standard solution with acetonitrile, followed by HPLC-FLD analysis. The linearity was determined using linear regression analysis by plotting peak area response (*y*) versus concentration (*x*). The obtained the following linear Equation (1) with a correlation coefficient (*R*^2^) of 0.9999, indicating good linearity for OTA in its tested range of 0.625 to 50 ng/mL.

*y* = 248,382*x* + 2723 (1)

For evaluating the method sensitivity, LOD and LOQ were measured as the lowest concentration of OTA that produce chromatographic peaks with signal-to-noise ratios of 3 and 10, respectively. The results show that relatively low LOD (0.4 ng/mL) and LOQ (1.25 ng/mL) were obtained for OTA, which were both lower than the maximum residue limits established by the European Union and other organizations.

Intraday reproducibility and interday variability were tested to evaluate the precision of the established HPLC-FLD method by performing repeat analyses of spiked malt and ginger samples with 15 μg/kg of OTA and was expressed as the relative standard deviation (%RSD) of relative peak area of OTA as the assessment index. The intraday reproducibility was assayed by six consecutive injections of OTA working solution on the same day, while the interday variability was assessed from six injections of OTA on six different days. The three RSD values were all lower than 8.0%, indicating good precision of the established method.

Accuracy was evaluated through recovery experiments. For this evaluation, the OTA-free raw malt and dried ginger sample powders were spiked with OTA at high (25 μg/kg), medium (15 μg/kg), and low (10 μg/kg) concentration levels, respectively, followed by the preparation and IAC clean-up and HPLC-FLD analysis. The recovery rate (%) was calculated according to the following Equation (2) to calculate the corresponding RSD.

Recovery (%) = (measured content/spiked amount) × 100 (2)

Results showed that the average recovery rates were from 70% to 86% with RSDs lower than 5%, which were in accordance with the requirements of the European Union and other organizations.

The above data have demonstrated that the validated HPLC-FLD method was satisfactory for the sensitive and accurate determination of OTA in raw malt and dried ginger samples.

### 2.2. HPLC-FLD Chromatograms of OTA in Tested Samples for Reuse of IAC

**Figure 1:**
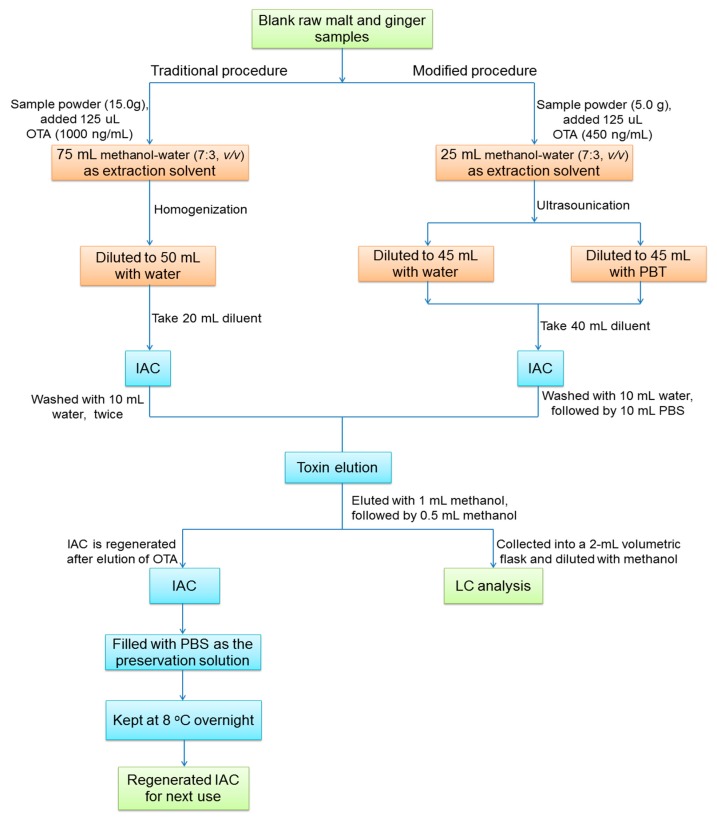
Sample preparation and immunoaffinity column (IAC) regeneration for ochratoxin A (OTA) analysis.

**Figure 2:**
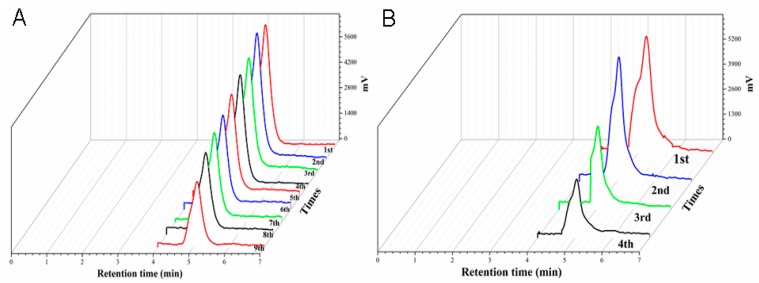
High performance liquid chromatography-fluorescence detection (HPLC-FLD) chromatograms of OTA in the spiked (**A**) raw malt and (**B**) dried ginger samples after reuse of IACs for different times.

According to the modified sample preparation with water as the diluent and IAC regeneration procedure in Figure 1, 11.25 μg/kg of OTA was added to malt and ginger samples, respectively. The HPLC-FLD chromatograms of OTA after reuse of IAC for purification and enrichment were shown in Figure 2. It could be seen that the OTA peak heights were all gradually decreased with increasing the regenerated and used times of IACs.

A recovery rate over 70% of the added OTA at the spiking levels is considered acceptable in accordance with AOAC method performance standards. As was expressed in Figure 2A, where the IAC was used up to nine times, the peak height and area of OTA were too low to be accurately quantified, and the calculated recovery rate did not reach the acceptable level of 70%. When IACs were used for 1, 2, 3, 4, 5, 6, 7, 8, and 9 times, 12.8, 12.4, 11.3, 10.6, 10.4, 9.2, 9.0, 8.1, and 6.8 μg/kg of OTA were detected from the spiked malt samples, respectively. While, for the spiked ginger samples in Figure 2B, the calculated recovery rates were lower than 70% when the IACs were used for more than three times. When IACs were used for 1, 2, 3, and 4 times, 12.8, 11.7, 7.7, and 6.1 μg/kg of OTA were measured from the spiked ginger samples, respectively. The above findings have indicated that when the used times of IAC was up to nine times for the spiked raw malt and four times for the spiked dried ginger samples; the detected contents of OTA exhibited a 50% reduction with recovery rates less than 70%. Therefore, the IAC can be used eight times for raw malt and three times for dried ginger regarding the clean-up and enrichment of OTA. Possible reasons for this might be that the activity of anti-OTA antibodies on the IAC and the antigen–antibody binding capacity were influenced or lowered due to different matrix types, sample preparation methods, pH values of sample solutions, and other factors, which would be taken into consideration in the next parts.

### 2.3. Effect of Matrix Type on IAC Regeneration

Raw malt and dried ginger are both complex systems of multicomponents belonging to different groups or classes, which will result in unpredictable influences on the activity of anti-OTA antibodies, antigen–antibody reaction, as well as the clean-up efficiency and recovery rate of IAC for target analytes. When the complicated components and possible interferences were passed through the IACs, they might bind with the anti-OTA antibodies to lower the recovery rates and reduce the regeneration times of the columns. As shown in Figure 2, the IACs could be regenerated and used eight times for the spiked raw malt samples and three times for the spiked dried ginger samples with the recovery rate more than 70% for OTA, which might be due to the differences of compositions and physical/chemical characteristics of the two matrices. Therefore, specific analysis should be conducted according to the differences of matrix types when studying the reuse of IAC for OTA analysis.

### 2.4. Effect of Sample Preparation Procedure on IAC Regeneration

**Figure 3:**
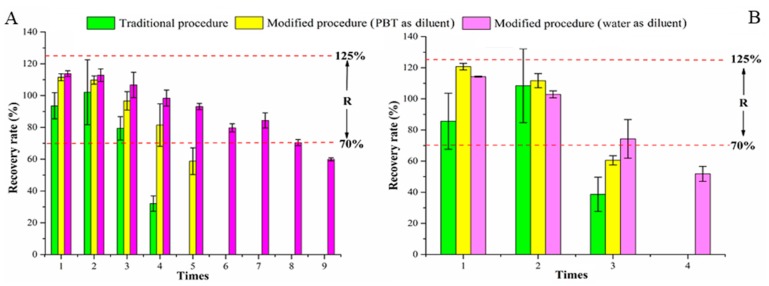
The OTA recovery and reuse times of IACs in the spiked (**A**) raw malt and (**B**) dried ginger samples using the traditional procedure and modified procedures with water and PBT as the diluents.

Primary experiments have shown that when introducing the traditional procedure recommended in Chinese Pharmacopoeia (2015 edition), the recovery rates of OTA together with the regeneration and reuse times of IACs for raw malt and dried ginger samples were all low, which were not satisfactory for accurate and economic analysis. One of the key factors might be the selection of the diluent before IAC clean-up. Therefore, some modifications were performed on the basis of the traditional procedure with water or phosphate-buffer tween (PBT) as the diluent, respectively, the sample preparation procedure has been shown in Figure 1. After the test, the OTA recovery and IAC reuse times were shown in Figure 3 when using three different preprocessing methods.

Regarding the traditional procedure recommended in Chinese Pharmacopoeia (2015 edition), it could be found that the IACs could be regenerated and used three and two times for the spiked raw malt and dried ginger samples, respectively. Otherwise, the recovery rates of OTA would be less than 70%. For the modified procedure with PBT as the diluent, the IACs could be regenerated and used four and two times for the two matrix types, respectively, with recovery rates more than 70%. While, in regard to the modified procedure with water as the diluent, it could be seen that the IACs could be regenerated and used up to eight and three times for the two matrix type, respectively, without losing the binding properties of anti-OTA antibodies. When the IACs were used for the ninth and fifth time, the recovery rates were 59.90% and 51.80%, respectively, which were not acceptable. The above findings have confirmed the influences of sample preparation procedure, especially the diluent on IAC regeneration and reuse for OTA clean-up and enrichment in tested sample matrices. In conclusion, the modified sample preparation procedure with water as the diluent was preferred for the regeneration and reuse of IACs for OTA clean-up in complex raw malt and dried ginger samples and the reuse times of IAC have been increased significantly.

### 2.5. Effect of pH Value of Sample Solution on IAC Regeneration

The pH value of sample solution was reported to express great influences for antigen–antibody binding on the IACs [22], and IAC instructions for use also required sample solutions through IAC finally with pH 6–8. Therefore, the effects of solution pH on reuse of IAC were investigated.

Primary experiments have indicated that the pH values of the final sample solution using the modified procedure with water as the diluent were in the range of 5 to 6. Herein, the sample solution pH values were adjusted to 5–6, 6–7, 7–8, and 8–9 with 1 M HCl or 1 M NaOH before IAC purification to investigate the effect of sample pH on regeneration and reuse times of IACs.

As could be seen in Table 1, when the pH of sample solutions were adjusted to 5–6 and 6–7, the IACs could be used only one time for raw malt and dried ginger samples with recovery rate more than 70%. After the pH of sample solution was adjusted to 7–8, the reuse times of IACs were significantly increased to eight times for the spiked raw malt sample and three times for the spiked dried ginger sample with acceptable recovery rates (>70%) for OTA. However, when the pH values were continuously increased to 9–10, the reuse times of IACs were rapidly lowered to two times for both the spiked raw malt and dried ginger sample solutions. 

The above-findings have shown that acidic or alkaline sample solutions of raw malt and dried ginger would significantly lower the regenerated and reused times of IACs for OTA. Neutral solution at pH 7–8 was preferred and helpful for the regeneration and reuse of IACs to lower the detection cost. In summary, a too high or too low pH value of CHM solution would lower the binding and clean-up properties of IACs, as well as the regeneration and reuse of IACs for OTA detection. Therefore, taking regeneration and reuse of IACs into consideration, the pH values of CHMs solution should be adjusted to 7–8.

### 2.6. Effect of Other Factors on IAC Regeneration

The regeneration of IACs might be affected by a variety of other factors, such as recovery interval of antibodies on the IACs, air exposure, eluting solvent for OTA, and the preservation solution used for IACs. Repeat experiments showed that the IACs could maintain good performance for multiple uses and clean-up of OTA, although using a short time interval for the recovery of OTA after each use. Exposure of IACs in air for four hours and eluting OTA with methanol had little effect on the regeneration of IACs. In addition, being filled with phosphate-buffered saline (PBS) solution as the preservation solution prior to the regeneration was suitable for the reuse of IACs.

## 3. Discussion

Although some pretreatment techniques and new analytical technologies [23,24,25,26] have emerged for mycotoxin detection, they are all in the developing stage. As a traditional and classical pretreatment tool, IACs are the most popular method for the clean-up of a target molecule in complex matrices prior to chromatographic analysis. But, the high cost has limited their wide application in practice. The regeneration of IACs for multiple reuses is an important and economical measure. As far as we know, this is the first attempt on the regeneration and reuse of IACs for OTA clean-up in complex raw malt and dried ginger samples prior to HPLC-FLD detection.

This study has proven that matrix type of CHMs, sample preparation procedure, sample extraction pH, together with other minor factors all exhibited effects on the regeneration of IACs regarding reuse for OTA clean-up. By regeneration, the IACs could be used up to eight and three times for the spiked raw malt and dried ginger samples, respectively, with a recovery rate over 70% for OTA. Using the modified sample preparation procedure, with water as the diluent, and adjusting the solution pH of raw malt and dried ginger to 7–8, the regeneration and reuse times of the IACs for OTA significantly increased, with all recoveries in an acceptable range. In addition, PBS is an easy-to-get and well-proven preservation solution for IACs before its regeneration for next use.

## 4. Conclusions

This is the first study on the regeneration and reuse of IACs for OTA clean-up in complex CHMs, providing a green, economical, and efficient tool for the analysis of a large number of samples at low cost. This study demonstrated that IAC could be regenerated and utilized for some CHMs by selecting appropriate sample preparation methods, adjusting solution pH, and adding preservation solutions for IAC column. When using raw malt and dried ginger as the tested matrices, IAC could be used up to eight times and three times by regeneration. Therefore, the corresponding experimental cost was also reduced to one-eighth and one-third of the original cost. This development is of great significance for extending the application of IAC columns and HPLC analysis of OTA and more mycotoxins in foods, CHMs, and other matrices.

## 5. Materials and Methods

### 5.1. Chemicals and Solution Preparation

OTA standard (1 mg OTA solid powder) was purchased from Pribolab (Singapore), OchraTest^TM^ IACs were bought from Huaan Magnech Bio-Tech Co., Ltd. (Beijing, China). HPLC-grade methanol and acetonitrile were obtained from Fisher Scientific (Fair Lawn, NJ, USA). Tween-20, Na_2_HPO_4_, and KCl were purchased from Sinopharm Chemical Reagent Co., Ltd. (Shanghai, China). NaH_2_PO_4_ and KH_2_PO_4_ were purchased from Xilong Chemical Industry Co., Ltd. (Beijing, China). Other reagents and chemicals were of analytical grade and acquired from Beijing Chemical Works (Beijing, China); Wahaha purified water (Wahaha Group Co., Ltd, Hangzhou, China) was used.

PBS solution was prepared by dissolving 4 g NaCl, 0.1g KCl, 45 g Na_2_HPO_4_, and 0.1 g KH_2_PO_4_ in 500 mL of water with the pH being adjusted to 7.4 with 0.1 M HCL. PBT solution was prepared according to the following procedure; 8.5 mL of 0.2 M NaH_2_PO_4_ solution and 91.5 mL of 0.2 M Na_2_HPO_4_ solution were mixed into for preparing 0.2 M phosphate-buffer (PB) solution. Then, 25 mL of 0.2 M PB was added into 475 mL of water to get 0.01 M PB. Finally, 10 mL of Tween-20 was added into 500 mL of 0.01 M PB to yield PBT solution (pH 7.8).

### 5.2. Apparatus

High performance liquid chromatography (HPLC) analysis of OTA was carried out on a Shimadzu LC-20AT HPLC system (Shimadzu, Kyoto, Japan) consisting of three LC-20AT pumps, a SIL20A autosampler, and a RF-10 AXL fluorescence detector (FLD). Chromatographic separation was achieved on an Agilent ZORBAX SB-C18 (4.6 × 250 mm, 5 μm) column (Agilent Technologies, Santa Clara, CA, USA) kept at 35 °C. An AB-135 Analytical Balances from Mettler Toledo (Switzerland), a VORTEX-5 vortex mixer (Haimen Kylin-Bell Lab Instruments Co., Ltd., Jiangsu, China), a PB-10 pH meter (Germany), and a KQ-25ODE CNC ultrasonic cleaning machine (Kunshan Ultrasonic Instruments Co., Ltd., Kunshan, China) were used through the experiments.

### 5.3. Liquid Chromatography and Fluorescence Detection Conditions

The mobile phase was consisted of 0.1% formic acid water–acetonitrile (40:60, *v/v*) at a flow rate of 1.2 mL/min, freshly prepared every day. The fluorescence detector was set at an excitation wavelength of 333 nm and an emission wavelength of 460 nm. The injection volume was 50.0 μL. Instrument operation, as well as data acquisition and processing, was performed via the LC solution ChemStation software (Shimadzu, Kyoto, Japan) on a personal computer.

### 5.4. Samples Collection

Malts are the mature fruits of the gramineous plant barley (*Hordeurn vulgare* L.) after being processed by germination and drying. Raw malts are used when the suitable temperature and humidity are maintained after the wheat grains are soaked in water. They are dried when the young shoots grow to approximately 0.5 cm. As a kind of CHM, malt expressed antibacterial, antivirus, antitumor, immunity enhancement activities, and so on [27]. Malt is also an important material for food, feed, and brewing products. However, it is highly susceptible to mildew and metamorphism to produce mycotoxins such as OTA, etc. [9]. Raw malt originated from Hebei province was purchased in Beijing TongRenTang Pharmacy Co., LTD. (Beijing, China) for this experiment.

Ginger is the fresh rhizoma of Zingiberaceae plant *Zingiber officinale* Rosc with antioxidant, anti-inflammation, and antimicrobial properties. It is also the main ingredient in many local cuisines because of its nutritional benefits and flavor. Like other CHMs, ginger can be easily infected by toxigenic fungi to produce mycotoxins including OTA [28]. Dried ginger samples originated from Sichuan province were purchased in Beijing TongRenTang Pharmacy Co., LTD. (Beijing, China) for this experiment after suitable drying.

Raw malt and fried ginger used for this article are all OTA-free and stored in the Institute of Medicinal Plant Development, Chinese Academy of Medical Sciences, Peking Union Medical College, Beijing, China.

### 5.5. Sample Preparation

The total scheme of sample preparation, together with IAC regeneration and reuse for OTA analysis has been presented in Figure 1.

#### 5.5.1. Traditional Procedure in Chinese Pharmacopoeia

The traditional procedure regarding sample preparation in Chinese Pharmacopoeia (volume IV, 2015 edition) [29] was firstly considered. A 15.0 g sample powder of raw malt and dried ginger, together with 3.0 g NaCl, was accurately weighed and placed in a homogenous bottle with the addition of 75 mL of methanol–water (70:30, *v/v*), and 125 μL of OTA standard (1000 ng/mL) solution. Then, the mixture was stirred at 11,000 rpm for 2 min followed by centrifugation for 5 min at 2500 rpm. Afterwards, 15 mL of the supernatant was exactly placed into a 50-mL volumetric flask and diluted with 35 mL of water. After mixing by vortex well for 30 s, the sample solution pH was adjusted to 7–8 and it was passed by a 0.45-μm syringe nylon filter for next IAC purification.

Twenty milliliters of the final filtrate was passed through an IAC at a flow rate of 3 mL/min and OTA in the spiked sample solutions was absorbed by OTA specific monoclonal antibody on the column. Then, the column was eluted with 20 mL of water until 2 to 3 mL of air was allowed to enter. OTA on the column was eluted with an appropriate amount (~1.5 mL) of methanol and collected into a 2-mL volumetric flask with the addition of methanol to the final volume. The mixture was vortexed for 20 s and filtered through a 0.22-μm syringe filter prior to HPLC-FLD analysis.

#### 5.5.2. Modified Procedure with PBT as the Diluent

The reported procedure [30] after some modifications was introduced for sample preparation. Five grams of triturated sample powder of raw malt and dried ginger, together with 1 g NaCl were accurately weighed, respectively, into a 50-mL centrifuge tube with the addition of 125 μL of OTA standard (450 ng/mL). After vortexing and mixing thoroughly with 25 mL of methanol–water (70:30, *v/v*), the mixture was ultrasonicated for 20 min and then centrifuged at 10,000 rpm for 5 min at 25 °C. An aliquot of 5 mL of the supernatant was transferred into another 50-mL centrifuge tube and 40 mL of PBT (2%, pH 7.8) was added for diluting. The extract was adjusted to pH 7–8. Then the mixed diluent was filtered through a 0.45-μm filter and 40 mL of the filtrate was passed through the IAC at a flow-rate of 3 mL/min. After the sample extract was passed through the column thoroughly, 10 mL of purified water and 10 mL of PBS were added in the turn at the same flow-rate to wash the IAC successively until 2–3 mL of air was pushed through the column. Definitive elution was achieved by adding 1.5 mL of methanol onto the column and flushing with pure air. The, additional methanol was added to yield the final volume of 2 mL. The diluted solution was then passed through a 0.22-μm filter and shifted to a vial for HPLC-FLD analysis.

#### 5.5.3. Modified Procedure with Water as the Diluent

The addition of a certain amount of phosphate and Tween-20 to sample solution may exhibit some influences on the antigen–antibody binding property on the IAC. Therefore, the performance of water as the diluent for sample pretreatment was also investigated. For this, the procedure was the same as that in Section 5.5.2 except water was used instead of PBT as the diluent prior to HPLC-FLD analysis.

### 5.6. Immunoaffinity Column Purification and Regeneration

Because of its high specificity and practicability, IAC has received continuous concern for OTA clean-up and enrichment in trace analysis. But, its high price has limited its application scope in practice. Herein, we attempted to regenerate and reuse the IACs for OTA analysis in complex malt and ginger samples. For regeneration, after the elution of OTA from the IAC, the column was filled with PBS as the preservation solution and stored overnight at 8 °C for continued use of the same sample on the following days. Correspondingly, the IAC was regenerated after each use and was reused for the corresponding test extracts. Multitime-regeneration and reuse of the IAC was stopped until the recovery rate was less than 70%. The reuse times of the IAC were determined and compared.

## Figures and Tables

**Table 1:** Effect of sample solution pH on IAC regeneration and reuse.

Sample	Reuse Times of IACs	pH = 5~6		pH = 6~7		pH = 7~8		pH = 9~10	
		Rec ^a^	RSD ^b^	Rec	RSD	Rec	RSD	Rec	RSD
**Raw malt**	1	75.8	1.1	96.8	2.5	113.8	1.6	97.3	4.9
	2	50.8	12.1	56.5	9.8	112.8	3.5	74.7	1.3
	3	- ^c^	-	-	-	106.7	7.5	68.9	2.2
	4	-	-	-	-	98.4	5.1	-	-
	5	-	-	-	-	93.1	2.1	-	-
	6	-	-	-	-	79.7	3.3	-	-
	7	-	-	-	-	84.4	5.6	-	-
	8	-	-	-	-	70.4	2.8	-	-
	9	-	-	-	-	59.9	1.6	-	-
**Dried Ginger**	1	98.9	4.1	101.3	0.5	114.3	0.3	99.7	0.1
	2	56.3	10.7	96.9	7.1	102.9	2.1	78.7	12
	3	-	-	45.4	29.9	74.3	16.7	26.6	28.2
	4	-	-	-	-	51.8	9.3	-	-

^a^ Recovery rate, ^b^ Relative standard deviation, ^c^ Not detected.

## References

[1] Calixto J.B. (2000). Efficacy, safety, quality control, marketing and regulatory guidelines for herbal medicines (phytotherapeutic agents). Braz. J. Méd. Biol. Res..

[2] Rizzo I., Vedoya G., Maurutto S., Haidukowski M., Varsavsky E. (2004). Assessment of toxigenic fungi on Argentinean medicinal herbs. Microbiol. Res..

[3] Kong W., Wei R., Logrieco A.F., Wei J., Wen J., Xiao X., Yang M. (2014). Occurrence of toxigenic fungi and determination of mycotoxins by HPLC-FLD in functional foods and spices in China markets. Food Chem..

[4] Mally A., Hard G.C., Dekant W. (2007). Ochratoxin A as a potential etiologic factor in endemic nephropathy: Lessons from toxicity studies in rats. Food Chem. Toxicol..

[5] Bayman P., Baker J.L. (2006). Ochratoxins: A global perspective. Mycopathologia.

[6] Visconti A., Pascale M.A., Centonze G. (2001). Determination of ochratoxin A in wine and beer by immunoaffinity column cleanup and liquid chromatographic analysis with fluorometric detection: Collaborative study. J. AOAC. Int..

[7] Joint FAO/WHO Expert Committee on Food Additives (2007). Evaluation of Certain Food Additives and Contaminants.

[8] Kong W.J., Xiao C.B., Ying G.Y., Liu X.F., Zhao X.H., Wang R.L., Wan L., Yang M.H. (2017). Magnetic microspheres-based cytometric bead array assay for highly sensitive detection of ochratoxin A. Biosens. Bioelectron..

[9] Xiao C.-B., Liu Q.-T., Dou X.-W., Yang M.-H., Kong W.-J., Wan L. (2016). Rapid detection of ochratoxin A in malt by cytometric bead array based on indirect competition principle. Chin. J. Anal. Chem..

[10] Yang Y., Wen J., Kong W., Liu Q., Luo H., Wang J., Yang M. (2016). Simultaneous determination of four aflatoxins and ochratoxin A in ginger after inoculation with fungi by ultra-fast liquid chromatography-tandem mass spectrometry. J. Sci. Food Agric..

[11] Wen J., Kong W., Wang J., Yang M. (2013). Simultaneous determination of four aflatoxins and ochratoxin a in ginger and related products by HPLC with fluorescence detection after immunoaffinity column clean-up and postcolumn photochemical derivatization. J. Sep. Sci..

[12] Creppy E.E. (2002). Update of survey, regulation and toxic effects of mycotoxins in Europe. Toxicol. Lett..

[13] Li F.Q., Ji R. (2003). Research progress on the relationship between ochratoxin A and human health. J. Hyg. Res..

[14] Zejli H., Goud K.Y., Marty J.L. (2018). Label free aptasensor for ochratoxin A detection using polythiophene-3-carboxylic acid. Talanta.

[15] Schaut A., Saeger S.D., Sergent T., Schneider Y.-J., Larondelle Y., Pussemier L., Blank R., Peteghem C.V. (2008). Liquid chromatographic methods for biotransformation studies of ochratoxin A. Biomed. Chromatogr..

[16] Goud K.Y., Kailasa S.K., Kumar V., Tsang Y.F., Lee S.E., Gobi K.V., Kim K.H. (2018). Progress on nanostructured electrochemical sensors and their recognition elements for detection of mycotoxins: A review. Biosens. Bioelectron..

[17] Shim W.-B., Kolosova A.Y., Kim Y.-J., Yang Z.-Y., Park S.-J., Eremin S.A., Lee I.-S., Chung D.-H. (2004). Fluorescence polarization immunoassay based on a monoclonal antibody for the detection of ochratoxin A. Int. J. Food Sci. Technol..

[18] You S.Z., Xu Y., Deng S.Z., Huang Z.B. (2009). Development of immunoaffinity column of anti-ochratoxin A monoclonal antibody. Food Sci..

[19] Boudra H., Morgavi D.P. (2006). Development and validation of a HPLC method for the quantitation of ochratoxins in plasma and raw milk. J. Chromatogr. B.

[20] Rhemrev R., Pazdanska M., Marley E., Biselli S., Staiger S. (2015). Automated aflatoxin analysis using inline reusable immunoaffinity column cleanup and LC-fluorescence detection. J. AOAC Int..

[21] Iha M.H., Mini C.A., Okada I.A., Briganti R.C., Trucksess M.W. (2017). The use of regenerated immunoaffinity columns for aflatoxins B1, B2, G1 and G2 in peanut confection. J. Chromatogr. A.

[22] Ip S.-P., Che C.-T. (2006). Determination of aflatoxins in Chinese medicinal herbs by high-performance liquid chromatography using immunoaffinity column cleanup. J. Chromatogr. Corunña.

[23] Cao J.L., Kong W.J., Yang M.H., Yin L.H., Wan L. (2013). Research progress on rapid detection methods of mycotoxins. Chin. J. Pharm. Anal..

[24] Cao J.L., Zhou S.J., Kong W.J., Yang M.H., Wan L., Yang S.H. (2013). Molecularly imprinted polymer-based solid phase clean-up for analysis of ochratoxin A in ginger and LC-MS/MS confirmation. Food Control.

[25] Yang X., Hu Y., Kong W., Chu X., Yang M., Zhao M., Ouyang Z. (2014). Ultra-fast liquid chromatography with tandem mass spectrometry determination of ochratoxin a in traditional Chinese medicines based on vortex-assisted solid-liquid microextraction and aptamer-affinity column clean-up. J. Sep. Sci..

[26] Freire L., Sant’Ana A.S. (2018). Modified mycotoxins: An updated review on their formation, detection, occurrence, and toxic effects. Food Chem. Toxicol..

[27] Yang T., Zeng Y.W., Xiao F.H., Pu X.Y., Du J., Yang S. (2007). Research progress of medicinal barley and its active substances. J. Chin. Crop Sci..

[28] Wen J., Kong W., Hu Y., Wang J., Yang M. (2014). Multi-mycotoxins analysis in ginger and related products by UHPLC-FLR detection and lc-ms/ms confirmation. Food Control.

[29] Chinese Pharmacopoeia Commission (2015). Chinese Pharmacopoeia.

[30] Zhang L., Dou X.W., Kong W.J., Liu C.M., Han X., Yang M.H. (2017). Assessment of critical points and development of a practical strategy to extend the applicable scope of immunoaffinity column cleanup for aflatoxin detection in medicinal herbs. J. Chromatogr. A.

